# Sulfurous Gases As Biological Messengers and Toxins: Comparative Genetics of Their Metabolism in Model Organisms

**DOI:** 10.1155/2011/394970

**Published:** 2011-11-10

**Authors:** Neal D. Mathew, David I. Schlipalius, Paul R. Ebert

**Affiliations:** ^1^School of Biological Sciences, University of Queensland, St. Lucia Campus, Brisbane, QLD 4072, Australia; ^2^Agri-Science Queensland, Department of Employment Economic Development and Innovation, EcoSciences Precinct, GPO Box 46, Brisbane, QLD 4001, Australia

## Abstract

Gasotransmitters are biologically produced gaseous signalling molecules. As gases with potent biological activities, they are toxic as air pollutants, and the sulfurous compounds are used as fumigants. Most investigations focus on medical aspects of gasotransmitter biology rather than toxicity toward invertebrate pests of agriculture. In fact, the pathways for the metabolism of sulfur containing gases in lower organisms have not yet been described. To address this deficit, we use protein sequences from *Homo sapiens* to query Genbank for homologous proteins in *Caenorhabditis elegans*, *Drosophila melanogaster*, and *Saccharomyces cerevisiae*. In *C. elegans*, we find genes for all mammalian pathways for synthesis and catabolism of the three sulfur containing gasotransmitters, H_2_S, SO_2_ and COS. The genes for H_2_S synthesis have actually increased in number in *C. elegans*. Interestingly, *D. melanogaster* and Arthropoda in general, lack a gene for 3-mercaptopyruvate sulfurtransferase, an enzym for H_2_S synthesis under reducing conditions.

## 1. Introduction

Despite initially being thought of only as toxic gases, hydrogen sulfide (H_2_S), nitric oxide (NO), and carbon monoxide (CO) are now recognized as important endogenously produced signalling molecules known as gasotransmitters. Wang describes gasotransmitters as small gas molecules that are membrane permeable, endogenously generated, and which have functions at physiologically relevant concentrations [1]. The first two gasotransmitters to be discovered were NO [2] and CO [3]. H_2_S was the third identified gasotransmitter [1, 4–7]. Like NO and CO, H_2_S is also a toxic air pollutant [8–10]. Sulfur dioxide (SO_2_) and carbonyl sulfide (COS) are gaseous toxins that only recently have been shown to be endogenously produced and to transmit biological signals [11]. In this paper, we will discuss the biology of the sulfur containing gasotransmitters and refer to their use as toxins. Our primary objective is to relate what is known in mammals to an understanding of the action of these compounds on invertebrate pests of agriculture. As such, we have augmented the paper with comparative bioinformatics of genes involved in the synthesis and catabolism of H_2_S, SO_2_ and COS. This will facilitate future detailed genetic studies into the mode of action of these gasotransmitters/sulphurous fumigants

The strongest evidence that SO_2_ and COS are gasotransmitters comes from their effect on smooth muscle cells. Dilation of vascular smooth muscle is caused by the endothelial release of vasodilator substances referred to as endothelium derived relaxing factor (EDRF) [2]. NO is a major mediator of EDRF-induced vasodilation [12], and H_2_S has been suggested as a secondary EDRF component [13]. However, EDRF causes hyperpolarization in smooth muscle cells, but neither NO nor H_2_S cause this effect. It has been suggested that EDRF contains more than one component that causes hyperpolarization, designated endothelium derived hyperpolarizing factors (EDHFs) [14, 15]. Both SO_2_ and COS are produced by the porcine coronary artery (PCA), and both have short half-lives of 1-2 seconds, similar to EDHF [11, 16, 17]. Therefore, SO_2_ and COS are potential candidates for EDHF [18]. 

Sulfur occupies a unique position in biology due to its ability to transfer electrons to and from substrates. Sulfur is a redox chameleon, with approximately ten different states of oxidation [19]. These range from negative two in thiols (RSH) to plus six in sulfate anions (SO_4_^2−^) and include fractional oxidation states such as −0.5, found in the disulphide radical anion (RSSR^−^) [20]. This unique chemistry allows sulfur to participate in an extensive range of redox events [21]. It also influences the catalytic and metal binding characteristics of the element [22] as well as the activity of the sulfurous gasotransmitters. For example, exposure to sulfur containing gases has a profound effect on cellular metabolic and redox systems [23–25].

We will discuss each of H_2_S, SO_2_, and COS, including their chemical properties, their metabolism, and their transport. We will also identify the orthologous sulfur metabolism and transport genes in the genetically tractable model organism *Caenorhabditis elegans *(*C. elegans*) as well as *Drosophila melanogaster* (*D. melanogaster*) and *Saccharomyces cerevisiae *(*S. cerevisiae*). *C. elegans *orthologues of mammalian genes involved in sulfation have recently been reviewed and will not be discussed here [26]. 

We propose that fumigants are effective poisons specifically, because they are able to disrupt endogenous gaseous signalling. This hypothesis has a close corollary—that fumigants or their close derivatives may have medically useful effects as modifiers of gasotransmitters at sublethal doses. This paper will facilitate future genetic investigation of these hypotheses.

## 2. Hydrogen Sulfide

H_2_S is a colourless, odorous, flammable, and water-soluble gas [27]. It is also highly toxic as evidenced by its use in the First World War as a chemical warfare agent [28]. H_2_S is also a significant air pollutant, particularly in sewerage treatment plants, where it can accumulate to dangerous levels [10]. The toxicity of these gases was initially presumed to be caused by the reversible inhibition of cytochrome c oxidase (COX), the terminal electron acceptor of the electron transport chain (ETC) [29]. 

It is now recognized that H_2_S has widespread biological roles. Thus, while H_2_S does inhibit COX at high concentrations of approximately 80 ppm similar to cyanide, at low concentrations H_2_S actually stimulates oxygen consumption [30]. H_2_S is found to efficiently compete with other electron donors. When H_2_S concentration is high in colonocytes, complex I of the ETC operates in reverse mode and accepts electrons from quinone in order to reduce NAD to NADH [31]. Inhibition of respiration caused by H_2_S is accompanied by a reversion of the ETC complex II [32]. 

Exposure to 150 ppm of H_2_S has been shown to induce a suspended animation like state in mice [33]. *C. elegans* acclimatized in 50 ppm of H_2_S results in thermotolerant and an increase in longevity [34]. Resistance to high temperatures in *C. elegans* often correlates with increased lifespan [35]. The lethal dose, 100% for *C. elegans *is 150 ppm of H_2_S. However, acclimatized *C. elegans* are able to survive being exposed to 500 ppm [36]. H_2_S also affects the cardiovascular [37], neural [4], digestive, respiratory, endocrine [38], and immune systems at physiological concentrations [39] (Table 1). H_2_S is endogenously produced during the metabolism of sulfur containing amino acids, in solution H_2_S dissociates to HS^−^ and H^+^ [40]. These biological activities have led to H_2_S being acknowledged as the third gasotransmitter following NO and CO [1, 4–7]. 

### 2.1. Synthesis of H_2_S

H_2_S is enzymatically generated via the desulfhydration of cysteine by two pyridoxal-phosphate (PLP) dependent enzymes cystathionine-*β*-synthase (CBS) [47] and Cystathionine-*γ*-lyase (CSE/CTH) [48]. As well as a PLP-independent enzyme 3-mercaptopyruvate sulfurtransferase (3MST/MPST) [49]. Both CBS and CSE are located in the cytosol [50], whereas 3MST is present in the cytosol and the mitochondria [51]. The biosynthetic pathway of H_2_S is dependent on the tissue location. CBS is the primary source in the brain [52] whereas CSE is the primary source of H_2_S in blood vessels [41]. Disruption of CSE results in an elevation of blood pressure [53]. 

**Figure 1:**
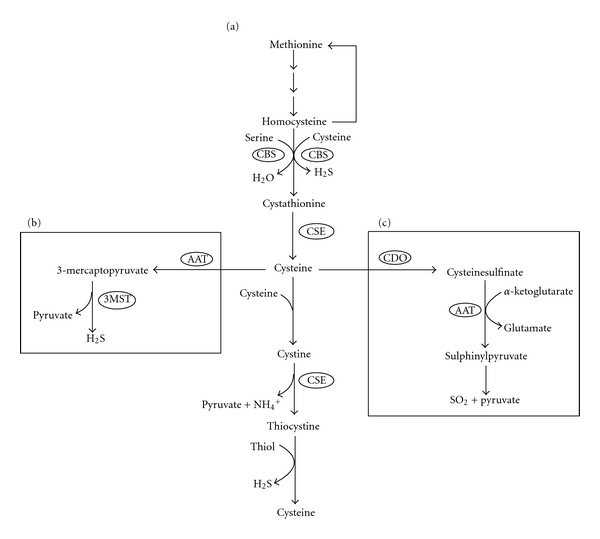
Metabolism of sulfur containing amino acids. (a) Homocysteine-dependent transsulfuration pathway that containing both cystathionine-*β*-synthase (CBS) and cystathionine-*γ*-lyase (CSE). Which is located in the cytosol and generates hydrogen sulfide (H_2_S). (b) H_2_S synthesis via aspartate aminotransferase (AAT) and 3-mercaptopyruvate sulfurtransferase (3MST), which occurs in the cytosol and mitochondria. (c) Catabolism of cysteine via cysteine dioxygenase (CDO) and AAT generates sulfur dioxide (SO_2_).

Both CBS and CSE affect not only the levels of H_2_S, but also the metabolism of sulfur containing amino acids and the redox state of the cell via their effect on the availability of glutathione (GSH). CBS and CSE are each involved in the homocysteine-dependent transsulfuration pathway. CBS catalyzes the first step in the catabolism of homocysteine to cystathionine, whereas CSE catalyzes the synthesis of cystathionine to cysteine (Figure  1(a)) [54, 55]. 

Availability of the sulfur containing amino acid cysteine is a critical factor in the synthesis of glutathione (GSH) [56, 57]. Roughly half of the intracellular GSH in the liver is derived from the transsulfuration pathway [58]. GSH and glutathione disulphide (GSSG) are the main thiol/disulphide couple involved in cellular redox maintenance (2GSH/GSSG) [59, 60]. H_2_S increases *γ*-Glutamylcysteine, which is a precursor to GSH and causes a recovery of cysteine transport [42, 43]. 

The heme in the CBS enzyme is redox-active and is capable of reversibly regulating the activity of the enzyme according to the redox state. Under reducing conditions cystathionine production is decreased by approximately 1.7 fold [54]. Whereas under oxidising conditions cystathionine production is increased between 1.6 and 2.1 fold [58]. Additionally, the expression of the CSE gene is also induced under oxidising conditions [61]. The redox responsiveness of these two pathways is likely important in order to maintain an appropriate intracellular glutathione pool [58, 62].

A third enzyme, 3MST, participates in a two-step pathway of H_2_S synthesis. Firstly, aspartate aminotransferase (AAT/ASAT/AspAT/GOT (Glutamic oxaloacetic transaminase)) [63] deaminates cysteine in the presence of *α*-ketoglutarate to generate 3-mercaptopyruvate and glutamate [64]. Secondly, 3-mercaptopyruvate is desulfhydrated to pyruvate and H_2_S by 3MST (Figure  1(b)). However 3MST activity is decreased under oxidative conditions, unlike CBS or CSE [65]. The inhibition results from oxidation of a catalytic cysteine in the active site of 3MST to sulfenate [66]. This inhibition helps to conserve cysteine in the cell, contributing to the maintenance of cellular redox homeostasis.

### 2.2. Catabolism of H_2_S

A paralogue of 3MST called Rhodanese (RHOD) is the principle enzyme involved in the detoxification of H_2_S in the mitochondria [67]. RHOD is also involved in the detoxification of cyanide [68]. H_2_S is rapidly oxidized to thiosulfate (S_2_O_3_^2−^) and then converted to sulfite (SO_3_^2−^) and sulfate (SO_4_^2−^) [69]. Vertebrate 3MST, which has 59% homology to RHOD can also potentially detoxify cyanide and H_2_S [67, 70].

### 2.3. *C. elegans*: Genes Involved in H_2_S Metabolism and Detoxification

#### 2.3.1. Cystathionine-*β*-Synthase (CBS)/Cysteine Synthase

**Figure 2:**
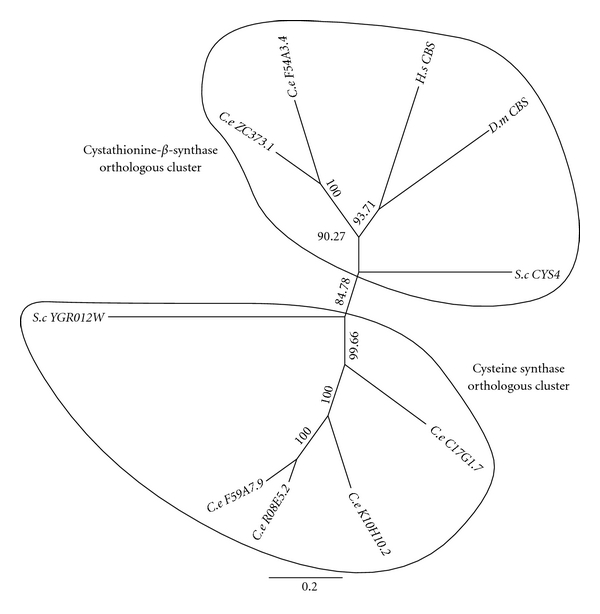
Phylogenetic tree analysis of cystathionine-*β*-synthase (CBS) and putative cysteine synthase (PCS). A blast (blastp) search of known *Homo sapiens* (*H.s*) proteins was undertaken against three different species, *Saccharomyces cerevisiae* (*S.c*), *Drosophila melanogaster* (*D.m*), and *Caenorhabditis elegans* (*C.e*) in the Genbank database. Identified sequences with significant expected value (≤1E−10) were used to generate a multiple sequence alignment (MSA) via ClustalW 2.1. The MSAs were then trimmed and used to produce an unrooted phylogenetic tree with 10,000 boostraps via Geneious 5.4.

The *S. cerevisiae *protein CYS4/YGR155W, *C. elegans *sequences ZC373.1 and F54A3.4, *H. sapiens *(CBS), and *D. melanogaster* (CBS/CG4840) form an orthologous cluster of sequences in the phylogenetic tree (Figure 2). Of these sequences, all but the *C. elegans* sequences have been characterized and shown to be CBS. There is also a somewhat more divergent yeast sequence (YGR012W) that defines a second orthologous cluster containing four *C. elegans* paralogues but no sequences from the other two organisms (Figure 2). YGR012W is a cysteine synthase located on the mitochondrial outer membrane [71].

#### 2.3.2. Cystathionine-*γ*-Lyase (CSE)

**Figure 3:**
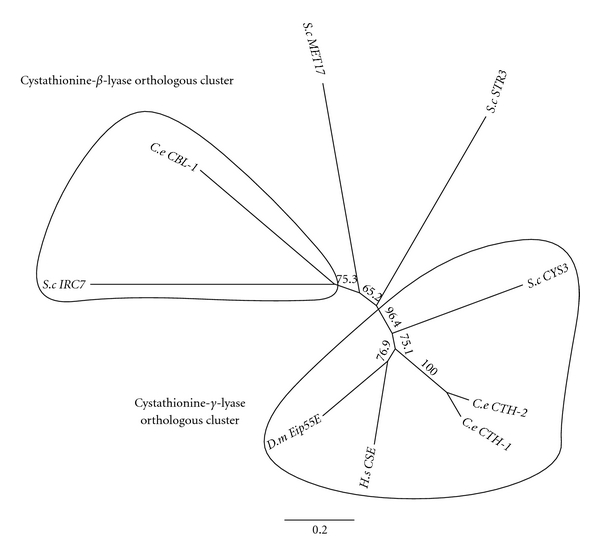
Phylogenetic tree analysis of cystathionine-*γ*-lyase and Cystathionine-*β*-lyase (CBL). F22B8.6 (CTH-1/CSE-1), ZK1127.10 (CTH-2/CSE-2), and C12C8.2 (CBL). See Figure 2 for details on the phylogenetic analysis.

The *S. cerevisiae *protein CYS3/YAL012W* D. melanogaster *Eip55E/CG5345, *H. sapiens *(CSE), *C. elegans sequences *CTH-2/ZK1127.10 and CTH-1/F22B8.6 form an orthologous cluster of sequences in the phylogenetic tree (Figure 3). *S. cerevisiae *protein MET17, catalyzes the reaction between O-acetylhomoserine and sulfide, leading to the production of homocysteine [72, 73]. *S. cerevisiae *proteins STR3 and IRC7 are cystathionine-*β*-lyase proteins not found in *H. sapiens*, which are involved in the biosynthesis of methionine [74, 75]. The *C. elegans *sequence CBL-1/C12C8.2 forms a second orthologous cluster with *S. cerevisiae* protein IRC7 (Figure 3).

#### 2.3.3. 3-Mercaptopyruvate Sulfurtransferase (3MST) and Rhodanese (RHOD)

**Figure 4:**
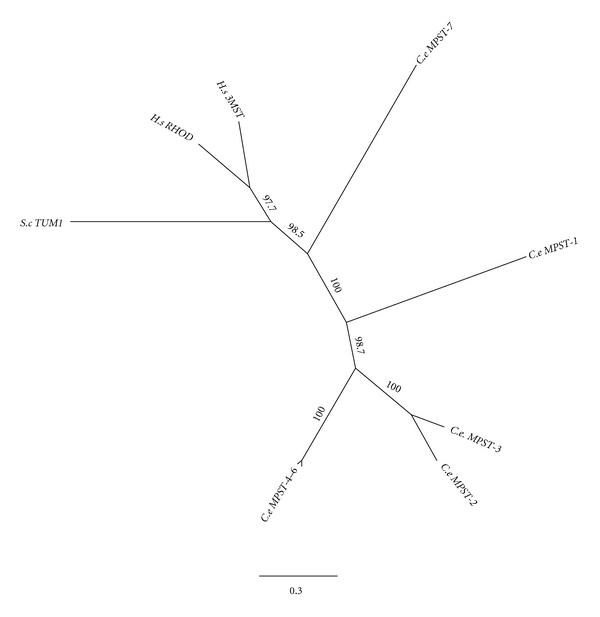
Phylogenetic tree analysis of 3-mercaptopyruvate sulfurtransferase (3MST/MPST). See Figure 2 for details on the phylogenetic analysis.

The *S. cerevisiae *protein TUM1/YOR251C, *H. sapiens *RHOD and 3MST as well as seven *C. elegans *paralogues MPST-1 through MPST-7 form an orthologous cluster of sequences in the phylogenetic tree (Figure 4). It is interesting to note that despite the gene being present in bacteria, yeast, nematodes, and mammals, no orthologous sequences exist in the *D. melanogaster *genome or in any Arthropoda sequences in Genbank.

#### 2.3.4. Aspartate Aminotransferase (AAT)

**Figure 5:**
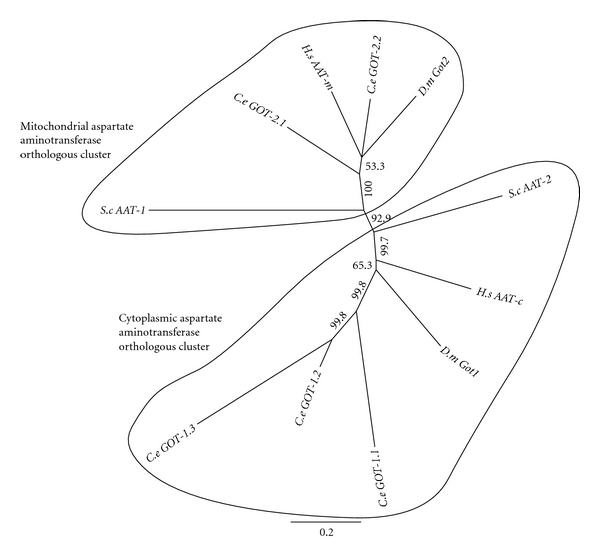
Phylogenetic tree analysis of aspartate aminotransferase (AAT/ASAT/AspAT/GOT (glutamic oxaloacetic transaminase)). See Figure 2 for details on the phylogenetic analysis.

The phylogenetic tree of aspartate aminotransferase sequences splits naturally into two clades. One clade contains the *S. cerevisiae *AAT1/YKL106W and *H. sapiens* AAT-m proteins, both of which are known to be located in the mitochondria. The other clade contains the *S. cerevisiae *AAT2/YLR027C and *H. sapiens *AAT-c proteins which are cytoplasmic [76]. The *D. melanogaster *protein GOT-2/CG4233 and *C. elegans* GOT- 2.1/C44E4.3 and GOT-2.2/C14F11.1 proteins fall in with the mitochondrial orthologues (Figure 5), which suggests that these proteins are mitochondrial as well. The D. melanogaster protein GOT-1/CG8430 and *C. elegans *GOT-1.1/T01C8.4, GOT-1.2/T01C8.5 and GOT- 1.3/C14E2.2 proteins fall into the cytoplasmic clade (Figure 5), which suggests that these proteins are cytosolic.

## 3. Carbonyl Sulfide

Carbonyl sulfide was first described in 1841 [77]. It is an air pollutant that also has been used as a fumigant [78, 79]. COS is also naturally present in the atmosphere, in water, soil, and plants [80]. COS is biologically generated in bacteria via the enzyme thiocyanate hydrolase, but this enzyme is not present in eukaryotes [81]. Interestingly, COS is detectable in both porcine coronary artery (PCA) and cardiac muscle and is able to induce arterial dilation [18]. As of the writing of this review the eukaryotic pathway of COS biosynthesis is not known. It has been shown, however that stimulation of PCA with acetylcholine causes an increase in synthesis of COS within the coronary artery. This suggests that muscarinic acetylcholine receptors (mAChRs) and not nicotinic acetylcholine receptors (nAChRs) are involved in regulating COS synthesis [18], because mAChRs but not nAChRs are found in the coronary artery [82].

COS is converted via *α*-carbonic anhydrase (*α*-CAH) to H_2_S and CO_2_. In eukaryotes, *α*-CAH is primarily responsible for pH regulation [83]. The enzyme is widely distributed in mammalian blood and tissue [84]. The toxicity of COS is mediated by H_2_S as inhibition of *α*-CAH activity decreased the toxicity of COS [85]. It is interesting to note that *α*-CAH activity can be inhibited via H_2_S [86]. After exposure to COS, the redox balance of the cell is disrupted and genes that respond to oxidative stress such as glutathione reductase and superoxide dismutase are upregulated [23]. The gene expression effect of exposure to COS is similar to that of phosphine exposure [87].

### 3.1. *C. elegans*: Genes Involved in COS Metabolism and Detoxification

The genes responsible for the synthesis of COS has not yet been identified, but it is known that *α*-carbonic anhydrase (*α*-CAH) is responsible for the conversion of COS to carbon dioxide and hydrogen sulfide. The *C. elegans α*-carbonic anhydrase gene family has been studied previously. It has six family members, two of which (CAH-3 and CAH-4) have been demonstrated to encode functional *α*-CAH enzymes (Table 2) [88]. Additionally *C. elegans*, *S. cerevisiae*, and *D. melanogaster *also contain a *β* class of CAH which is not found *H. sapiens *[89, 90]. 

## 4. Sulfur Dioxide

Like the gases mentioned previously, SO_2_ is a toxic air pollutant [91, 92]. It also has the distinction of being the oldest recorded chemical fumigant, as it was used by ancient Egyptians, Greeks, and Romans [93]. It was also used as a chemical warfare agent in a conflict between the Athenians and the Peloponnesians circa 431 B.C. [94] as well as during the Roman siege of Dura-Europos in 256 C.E. [95]. Sulfite, a dissociation product of SO_2_, is used as a preservative in beverages and food [96].

SO_2_ is likely to be a signalling molecule as it is produced endogenously from the metabolism of sulfur containing amino acids [97]. Additionally, SO_2_ has been found to produce biological effects at physiological concentrations [18], such as vasodilation in isolated rat aortic rings [98] and a decrease in blood pressure of male rats [99, 100]. For these reasons, SO_2_ has been suggested to be a gasotransmitter [98].

SO_2_ can also dissociate to its derivatives in solution, which may also be biologically active. For example, SO_2_ dissociates into sulfite (SO_3_^2−^) and bisulfite (HSO_3_^−^) in a 3 : 1 ratio in a neutral solution [101]. Furthermore, both SO_3_^2−^ and HSO_3_^−^ can be oxidized to SO_4_^2−^ via sulfite oxidase (SOX) [102]. Vasodilation via SO_2_, however, has been found to be much greater than dilation induced by SO_2_ derivatives [103]. It is, therefore, unlikely that activity attributed to SO_2_ is actually due to the action of derivative compounds.

### 4.1. Generation of SO_2_

SO_2_ is generated via two different pathways, one enzymatically and one nonenzymatic. The enzymatic metabolism occurs via catabolism of cysteine [49]. Cysteine catabolism to cysteinesulfinate is dependent upon cysteine dioxygenase (CDO) [97, 104]. CDO concentration is regulated by the availability of methionine and cysteine or protein [105]. Therefore, CDO is one of the enzymes that regulates free cysteine levels [106]. The key enzyme in the generation of SO_2_ is AAT, which is constantly being expressed (Figure  1(c)) [107]. However, expression of AAT is increased via glucocorticoids [11, 108]. AAT is expressed in cytosol and mitochondria [109]. Nonenzymatic generation of SO_2_ occurs at neutrophils as a result of oxidative stress, which causes the conversion of H_2_S to sulfite [110].

### 4.2. Toxicity

There is little information available on the mechanism of SO_2_ toxicity. It potentially involves oxidative damage caused by free radicals formed during sulfite oxidation [111]. Exposure to SO_2_ has been found to cause lipid peroxidation as well as increase the levels of enzymes that protect cells against oxidative damage caused by reactive oxygen species, including superoxide dismutase, glutathione peroxidase and catalase [112]. Exposure to SO_2_ also induces chromosomal aberrations, chromatid ex-changes and micronuclei formation, as shown in cultured human blood lymphocytes [113, 114]. Sulfite oxidase (SOX) is involved in oxidative detoxification of sulfite [102, 115]. Deficiency of SOX has previously been demonstrated to increase SO_2_, SO_3_^2−^, and HSO_3_^−^ toxicity [116, 117]. SOX activity has been shown to be significantly different in different mammalian tissues [118]. It is expected the oxidative damage caused by exposure to SO_2_ would be tissue specific. However it is found that exposure of SO_2_ caused nearly equal oxidative damage, suggesting that SO_2_ toxicity is systemic [119].

### 4.3. *C. elegans*: Genes Involved in SO_2_ Metabolism and Detoxification

Phylogenetic analysis was not carried out on the other proteins involved in the synthesis or breakdown of SO_2_ as no gene duplication had occurred, resulting in a simple one-to-one correspondence between nematode and human sequences. The human gene for cysteine dioxygenase (*cdo-1*) carries out the initial step in sulfur dioxide synthesis from cysteine. *C. elegans* has a single counterpart which is also called *cdo-1* (Table 3). The second step in sulfur dioxide synthesis is aspartate amino transferase, which is also used elsewhere in sulfur metabolism and is the subject of Figure 5. The final step in the synthesis of sulfur dioxide is the nonenzymatic decomposition of sulfinyl-pyruvate to sulfur dioxide and pyruvate. The oxidation of sulfur dioxide is carried out by SOX-1 in humans, the orthologous *C. elegans* gene is H13N06.4 (Table 3). 

## 5. Cross-Talk between Gasotransmitters

H_2_S and SO_2_ have been found to act synergistically with NO to enhance the vasorelaxant effect [100, 120]. The vasorelaxant effect of H_2_S in rat aortic rings can be decreased by removal of the endothelium, interruption of NO synthase or blocking of Ca^2+^-dependent K^+^ channels [121]. This suggests that NO and potentially EDHF contribute to the vasorelaxant effect of H_2_S. However, others have not observed synergistic vasorelaxation when H_2_S and NO treatments are combined but instead see a decrease in vasorelaxation [122]. This is likely due to reaction between H_2_S and NO to generate a nitrosothiol [123].

Synthesis of H_2_S is increased when exposed to NO due to the activation of the cyclic guanosine monophosphate pathway, which potentially increases the activity CSE [124]. NO also increases the expression of CSE which is involved in H_2_S synthesis [37]. It has also been found that metabolism of CO is also enhanced when exposed to H_2_S [125]. Additionally, NO and H_2_S can result in an increase in cysteine uptake [42, 126]. This increase in cystine uptake can then enhance the synthesis of H_2_S [127]. In contrast, H_2_S has been found to suppress NO synthesis in rats by down regulating the aortic L-arginine-dependent NO pathway [128]. Exposure to SO_2_ is found to increase NO pathway [129]. Despite the poor understanding of the cross-talk between gasotransmitters, it is clear that their functions are tightly integrated.

## 6. Storage, Release, and Transport

After the sulfur containing gases are endogenously produced, they can either be immediately released or stored for later release in response to a physiological signal. Unlike the short half-lives of COS and SO_2_, the half-life of H_2_S in mammalian plasma is about 30 minutes [130]. Sulfur from the sulfur containing gases can be stored in two forms, acid-labile sulfur or bound sulfane-sulfur. Exogenously H_2_S is absorbed and stored as bound sulfane-sulfur [131]. Sulfur is released from bound sulfane-sulfur by reduced glutathione and cysteine, or at pH higher than 8.4 [131, 132]. Acid-labile sulfur is generally found as iron-sulfur enzymes in the mitochondria. Sulfur is released from acid-labile sulfur at a pH of <5.4, with little or no H_2_S released at pH ≤ 6 [131]. 

Bound sulfane-sulfur is generally located in the cytoplasm though there is also evidence of long distance transport [131, 133]. One mechanism of transport has been determined in *Lucina pectinata* in which the heme group of hemoglobin I (HbI) binds and transports sulfide [97, 134]. In the C1 Hb from *Riftia pachyptila*, the sulfide is bound to zinc ions [135]. Neither HbI nor C1 Hb are found in *C. elegans*, though other globular proteins exits which could potentially bind to sulfide, providing a mechanism for long distance sulfide transport. 

H_2_S COS and SO_2_ are able to freely diffuse across the hydrophobic cellular membrane without facilitation via membrane channels [136–138]. Furthermore, no evidence of active membrane transport of H_2_S has been found [137]. This situation is analogous that of ammonia transport, which is also able to freely diffuse across the cellular membrane. Despite this ability, however, three distinct transport systems exist that actively transport ammonia across cell membranes [139].

## 7. Future Directions

The three sulfur containing gases H_2_S, SO_2_, and COS act as gasotransmitters in vertebrates. The primary bioassay that is used to study the three compounds is vasodilation, though metabolic arrest has also been demonstrated in the case of H_2_S and all three compounds are known to affect the redox state of cells. Though the compounds have barely been studied in invertebrates, H_2_S has been shown to mediate lifespan extension and heat tolerance in *C. elegans* [34] as well as desiccation tolerance in *D. melanogaster* [140]. Exposure of *C. elegans* to SO_2_ induces ovoviviparity [141], which is a stress response in *C. elegans* [142]. The toxicity of the chemicals when administered at concentrations greater than normal endogenous levels likely reflects their role as potent neurological and physiological signalling molecules. The toxicity has been exploited commercially through the use of these compounds as fumigants.

Characterisation of the roles of these molecules in the model organism, *C. elegans*, will facilitate the genetic analysis of their function and toxicology with benefits to be gained in agriculture and medicine. A first step toward genetic analysis is to determine the extent to which the metabolic pathways exist in the primary eukaryotic genetic model organisms; *S. cerevisiae*, *C. elegans*, and *D. melanogaster*. Our analysis reveals that all of the metabolic genes are present in yeast. Studies in this organism will be of limited value in understanding how the compounds act in a multicellular animal, however. *D. melanogaster* and indeed all insects are missing one of the metabolic genes altogether, which will limit the studies that can be considered in this species. The model system *C. elegans* contains all of the mammalian genes involved in the metabolism of the sulfur containing gases. *C. elegans* has an additional interesting property. Most genes for synthesis of H_2_S have been duplicated in *C. elegans* even though single genes exist in the other organisms. This situation likely reflects the stereotypic biology of *C. elegans* in which specific genes act in specific cell types to a greater extent than in other organisms. This may provide a research advantage as genetic manipulations may allow gasotransmitter signalling to be disrupted more specifically than in other organisms. 

*C. elegans* is ideal for the genetic investigation of gasotransmitter action and toxicity as the nematodes are cultured on agar medium which facilitates simultaneous exposure to dissolved chemicals and gases. *C. elegans* reproduces rapidly as a self-fertilizing hermaphrodite which facilitates the creation of mutant strains. There are also well-defined techniques for transformation and genetic manipulation of gene expression. Furthermore, the stereotypic development of *C. elegans* means the origin of each cell in the adult is known and the physiological role of each cell is reproducible. Because the organisms are transparent, the physiology of individual cells in the living organism also can often be studies microscopically using fluorescent probes.

Genetic analysis can be carried out in the “forward" direction, which refers to the traditional approach of mutagenesis of all genes in the genome followed by a screen for mutant individuals that exhibit a specific effect (e.g., resistance to H_2_S). Analysis then reveals the gene that was mutated and how the phenotype is mediated. Genetic analysis can also be carried out in the reverse direction, which refers to the molecular genetic approach of disrupting a characterised gene that ought to affect a process and then analysing the result. An example would be to individually suppress each CSE gene in *C. elegans* to see which of them result in phenotypes related to H_2_S synthesis. Genetic analysis can also be combinatorial, an approach that is greatly facilitated in genetic model organisms, which usually have large collections of characterised mutant strains that are distributed to researchers on request. An example of a combinatorial approach would be to determine the effect of H_2_S depletion on lifespan in strains carrying known longevity mutations. This would indicate whether the effect of H_2_S on lifespan is related to any previously described lifespan enhancing mutations. 

Major issues remain to be investigated regarding the roles of sulfur containing gases in biology, particularly in invertebrates. These include the possibility of unique roles of the gases within specific subcellular compartments, in specific tissues or at specific times during development. Genetic analysis can be used to identify interactions between gaseous signals and other signalling pathways as well as the influence of redox state on the activity of the gases. Understanding the mechanisms of action can also be used to identify novel fumigants or fumigation synergists of commercial importance. Model organism genetics can also be used for the identification and genetic manipulation of physiological parameters of medical significance that are controlled by the sulfur containing gases. Such physiological states include thermotolerance, desiccation tolerance, reversible metabolic arrest, and hypoxic preconditioning. All of these research targets can be addressed effectively and meaningfully in genetic model organisms such as *C. elegans*.

## Figures and Tables

**Table 1:** Effects of H_2_S.

Cause	Effect
Vasodilator	H_2_S like NO and CO, causes the opening the potassium adenosine triphosphate (K_ATP_) channels [37]
Apoptosis modulator	Via the activation of the mitogen-activated protein kinases (MAPK) pathway [41]
Protection against oxidative stress	Increases GSH synthesis and recovery of cysteine transport [24, 42, 43]. Scavenging of hydroxyl, oxygen and nitric oxide free radicals and reduces the accumulation of lipid peroxidation [44–46]
Neuromodulator	Enhances activity of *N*-Methyl-D-aspartic acid (NMDA) receptor and activates calcium channels, which regulates synaptic transmission in neurons [4]

**Table 2:** A-CAH *C. elegans* genes.

Sequence name	Gene name	Predicted/confirmed function	% Identity	*E* value
F54D8.4	CAH-1	*Homo sapiens* orthologs—CAH related protein 10 (partially confirmed via cDNA)	77/213 (36%)	1*e* − 41
D1022.8	CAH-2	*Homo sapiens* orthologs—CAH related protein 10 (confirmed via cDNA)	106/288 (37%)	7*e* − 60
K05G3.3	CAH-3	*Homo sapiens* orthologs—CAH7 isoform 1 (confirmed via cDNA)	96/262 (37%)	2*e* − 46
R01E6.3	CAH-4	*Homo sapiens* orthologs CAH-2 (confirmed via cDNA) functioning CAH	71/234 (30%)	1*e* − 30
R173.1	CAH-5	*Homo sapiens* orthologs—CAH7 isoform 1 (confirmed via cDNA)	96/261 (37%)	3*e* − 49
T28F2.3	CAH-6	*Homo sapiens* orthologs—CAH7 isoform 1 (partially confirmed via cDNA)	76/246 (31%)	1*e* − 36

**Table 3:** Other *C. elegans* genes involved in SO_2_.

Sequence name	Gene name	Predicted/confirmed function	% identity	*E* value
H13N06.4	SUOX-1	*Homo sapiens* orthologous (confirmed via cDNA)	230/471 (49%)	3*e*−160
F56F10.3	CDO-1	*Homo sapiens* orthologous (confirmed via cDNA)	87/176 (49%)	1*e*−67
